# Relationship between genotypes of the Duffy blood groups and malarial infection in different ethnic groups of Choco, Colombia

**Published:** 2012-09-30

**Authors:** Lina Gonzalez, Jorge Vega, Jose L Ramirez, Gabriel Bedoya, Jaime Carmona-Fonseca, Amanda Maestre

**Affiliations:** a Universidad de Antioquia, Medellin, Colombia

**Keywords:** Duffy blood-group system, ethnic groups, malaria, *Plasmodium falciparum*, *Plasmodium vivax*, genotypes, PCR-RFLP, Colombia

## Abstract

**Introduction:**

The negative homozygous condition for the Duffy blood group (Fy-/Fy-) confers natural resistance to *Plasmodium vivax* infection. Studies carried out in pursuing this direction in Colombia are scarce.

**Objective:**

To describe the relationship between Duffy genotypes in three ethnic communities of La Italia (Chocó) and malarial infection.

**Methods:**

This is a descriptive, cross-sectional study in symptomatic and asymptomatic subjects with malaria. Sample size: Afro-Colombians 73; Amerindian (Emberá) 74, and Mestizo, 171. The presence of *Plasmodium* infection was assessed by thick smear and the status of the Duffy gene was studied by PCR and RFLP to help identify changes to T-46C and A131G which originate the genotypes T/T, T/C , C/C and G/G, G/A, A/A.

**Results:**

Infection by *Plasmodium* was detected in 17% of cases with 62% due to *P. falciparum* and 27% due to *P. vivax*. Duffy genotypes were significantly associated with ethnicity (*p*= 0.003). Individuals with the C/C, A/A diplotypes were exclusively infected by *P. falciparum*, whereas the other diplotypes were infected with either of the species. In the Amerindian and Mestizo populations, the frequency of the T-46 allele was 0.90-1.00, among Afro-Colombians this was 0.50, the same as with the C allele and with an absence of heterozygous. At locus 131, the maximum frequency of the G allele was 0.30 in Amerindians and the maximum of the A allele was 0.69 in Afro-Colombians.

**Conclusions:**

In the Amerindian and mestizo populations studied, there was a predominance of the allele T-46 (FY+) but this was not observed with the *P. vivax *infection*.* *P. vivax* was ruled out in all FY- individuals.

## Abstract (es)

**Introducción:**

La condición homocigótica negativa del grupo sanguíneo Duffy ((Fy-/Fy-) confiere resistencia natural a *Plasmodium vivax.* En este sentido, los estudios realizados en Colombia son escasos.

**Objetivo:**

describir la relación entre los genotipos Duffy en tres comunidades étnicas de La Italia (Chocó), y la malaria.

**Métodos:**

estudio descriptivo, transversal con individuos sintomáticos o asintomáticos de malaria. Tamaño muestral por etnia: afrocolombiana 73; amerindia 74, mestiza 171. La infección plasmodial se estudió por gota gruesa y el estado del gen Duffy por PCR y RFLP con el fin de identificar los cambios T-46C y A131G que originan los genotipos T/T, T/C , C/C y G/G, G/A, A/A.

**Resultados:**

se encontró infección plasmodial en 17% con 62% por *P. falciparum* y 27% por *P. vivax*. Se halló relación entre genotipos Duffy y etnia (*p*= 0.003). Los individuos con diplotipos C/C,A/A se infectaron exclusivamente con *P. falciparum*, mientras que otros diplotipos por las dos especies. En amerindios y mestizos la frecuencia del alelo T-46 fue 0.90-1.00, en afrocolombianos fue 0.50, al igual que el alelo C, con ausencia de heterocigotos. En el locus 131 la máxima frecuencia del alelo G fue 0.30 en amerindios y la máxima del alelo A fue 0.69 en afrocolombianos.

**Conclusiones:**

En las poblaciones amerindia y mestiza estudiadas predomina el alelo T-46 (FY+), pero no predomina en ellas la infección por *P. vivax.* Ningún individuo FY- tuvo infección por *P. vivax.
*

## Introduction

In humans, the presence of certain polymorphisms in genes or proteins expressed on the plasma membrane of red blood cells is related to innate resistance for malarial infection^1^. Sickle cell anemia, deficiency of glucose-6-phosphate dehydrogenase (G6PD ) and α and β thalassemia are the result of polymorphisms that confer partial protection against infection by *Plasmodium falciparum* ^1^. Moreover, the Duffy negative phenotype (FY-) confers resistance to blood infection by *P. vivax*, i.e. absence of the protein does not allow merozoite binding to the surface of the erythrocyte membrane^1^. The erythroid polymorphism Fya and Fyb of the Duffy protein is a result of the substitution of an amino acid, glycine, asparagine at position 42 of the extracellular domain of the polypeptide , whereas the absence of expression of Fy is a result of replacing cytosine with thymine at the promoter region of the gene. These changes generate four major phenotypes: Fya+b+, Fya+b-, Fya-b+ and Fya-b-^2^.

Despite the important clinical and epidemiological evidence regarding protection against *P. vivax* in Duffy negative individuals, several studies have suggested the presence of infection with this species in the absence of protein expression^3^ ^-^ ^8^. This could be explained by the fact that Fyb is the ancestral allele and Fya expression results in decreased efficiency of the binding of *P. vivax* and, therefore, in decreased susceptibility without complete protection against invasion^3^ ^,^ ^9^ ^,^ ^10^. This phenomenon could hypothetically result in asymptomatic parasitemia.

It has been postulated that *P. vivax* has exerted pressure for the selection of the C allele (Fybnull) since in Africa a high correlation was confirmed between the absence or low endemicity of malaria from *P. vivax* and the high prevalence of the negative Duffy phenotype in the native population^1^ ^,^ ^11^ ^-^ ^14^. In Colombia there are few studies with the Duffy blood group as a genetic marker to identify Afro-Colombian individuals^15^, and there are even fewer studies in which malaria is associated with the different phenotypes and genotypes of the Duffy gene. In 1994, Montoya *et al*.^16^, found *P. vivax* infection in Fy- subjects (assessed by agglutination) with a frequency of 8.9%.

Recently, the analysis of the infecting species in an African-Colombian population from the Pacific Coast region has confirmed the predominance of the *P. falciparum* infection, a fact that has been attributed to the absence of the Duffy receptor in this ethnic group^17^. Other researchers have shown a high frequency of Fy- individuals among the inhabitants of this region of the country^18^.

This research had the objective of understanding the molecular characteristics of the Duffy gene in three ethnic communities (Black, Amerindian and Mixed or mestizo) who are inhabitants of the same eco-epidemiological area of highly endemic malaria and explore the relationship with the presence of infections by *P. vivax* and *P. falciparum*. The inclusion of both symptomatic and asymptomatic individuals with malaria was undertaken as a first approach to identifying a relationship with different Duffy genotypes and the possibility of partial protection against infection by *P. vivax*. 

## Materials and Methods

The research was carried out in the town of San José del Palmar in the La Italia district of the state of Chocó (4° 51' NL, 76° 18' WL). La Italia has a population of four thousand people and has an ethnic composition of 2,500 Afro-Colombian individuals, 1,000 Afro-mestizos (resulting from the mixture of two ethnic groups) and 500 Amerindians (Embera, mostly residents of the Campoalegre reservation).

### Study Population and Sample: 

A descriptive, cross-sectional and prospective design was applied to determine the prevalence of the Duffy antigen in the three ethnic groups and its association with the differing infectious species of *Plasmodium*. Based on a confidence level of 95%, and a sampling error of 5%, the prevalence of the negative Duffy antigen is 0.95 for Afro-Colombians, 0.05 for indigenous natives and 0.16 for mestizos. Representative samples were calculated for each group as follows: 71 Afro-Colombians, 64 Amerindians, and 171 Mestizos^15^ ^,^ ^19^ ^-^ ^22^. Criteria for inclusion were sequentially: volunteer subjects over 1 year of age who claimed Afro-Colombian heritage by "culturally belonging to the black community by self-report", Amerindian if "identifies or recognizes himself as belonging to a particular ethnic group with a cultural tradition predating the Spanish Conquest and that lives in the community, i.e. in the territory occupied by your community or group" and Mestizos if "recognizes himself as coming from a mixture of heritages, i.e. Europeans, Africans and Indigenous persons since 1492 in Hispanic America"^23^. The only exclusion criterion was withdrawal of informed consent to participate.

### Clinical and parasitological evaluation: 

Volunteers were clinically evaluated by physicians experienced in the diagnosis of malaria. Symptoms and signs associated with malaria were recorded according to the protocol of the World Health Organization for the definition of probable clinical cases. An individual was considered asymptomatic who in the two weeks prior had no clinical signs and symptoms of malaria to report at the time of the examination. The diagnosis of *Plasmodium* infection was performed with thick and thin blood smears prepared from capillary blood obtained by pricking the pad of a finger. The slides were stained with Giemsa or Field, and examined by expert personnel. The parasitemia was calculated based on a constant of 8,000 leukocytes/mm. Samples were considered negative after examining 200 microscopic fields with 100x magnification.

### Genotyping of Duffy antigen: 

The state of the Duffy gene was determined in all individuals, but the Duffy phenotype was not analyzed. Starting with peripheral white blood cells or epithelial cells from oral mucosa, DNA was obtained through "Salting out" or use of phenol-chloroform. The DNA obtained was precipitated and stored at -20° C until it was used.

The identification of the different alleles of the Duffy gene was done based on the methodology published by Tournamille *et al.* ^2^ which allows for an evaluation of the state of the positions -46 and 131 of the gene in order to identify T-46C and A131G substitutions responsible for the different alleles. Briefly, specific primers were applied to amplify a fragment of 1.0 kb of the Duffy gene. The product of this first reaction was used as template for: (a) a second amplification reaction with new primers to discriminate between Fy+ and Fy-; and, (b) in the case of Fy+, a second amplification was performed with specific primers to define the polymorphism of the genotype that originates the phenotypes Fya+Fyb+, Fya+Fyb- and Fya-Fyb+. The products of the second amplification were subjected to an analysis of the restriction fragments (RFLP in the English acronym: restriction fragment length polymorphism)^2^, with Sty 1 and BshNI (Fermentas(r)).

The digestion products were observed on 15% polyacrylamide gel stained with ethidium bromide. The size of the fragments were assessed using the Quantity One software program (Bio-Rad) as follows: bands 62, 77 and 82 base pairs (bp) correspond to Duffy positive (Fy+); bands 12, 62, 65 and 82 bp were interpreted as Duffy negative (Fy -); bands 86 and 73 bp are Fya+b-; bands 159, 86 and 73 bp are Fy a+b+; and a band of 159 pb is Fy a-b+. Positive and negative controls were samples of typing now standardized in the laboratory of the Group Health and Community.

### Statistical analysis: 

Qualitative and quantitative variables were analyzed. Qualitative variables were analyzed by chi square test with the EpiInfo 6.0 software. Obtaining genotypic and allelic frequencies and tests of neutrality and genetic structure were done using the "Genepop" program, version 3.1. The generation of the more likely haplotypes was done ​​with the "Arlequin" program, version 2.000. The population structure was estimated with the genetic distance of Cavalli-Sforza through use of the Neighbor-Joining algorithm (UPGMA version 3.572c).

### Ethical considerations: 

The research complied with the requirements of Resolution 008430 (1993) from the Colombian Ministry of Health, the Nuremberg Code 1947 and the Helsinki Declaration of 1987 and was approved by the Ethics Committee of the Medical Research Institute, School of Medicine, Universidad de Antioquia. The design of the proposal met with the verbal approval of the local governor of the indigenous community and each individual, or his guardian, who agreed to participate, signed or left their digital fingerprint on the informed consent form. Individuals diagnosed with malaria received, at no cost, the treatment recommended by Colombian standards. This research was considered to be of minimal risk.

## Results

### Description of the study population: 

In total, 320 volunteers were studied. The ratio between males and females was 131/189. The ethnic distribution was: 73 Afro-Colombians (23%), 74 Native Indians (23%) and 173 (54%) Mestizos. Table 1 shows the general characteristics of the population.

**Table 1:**
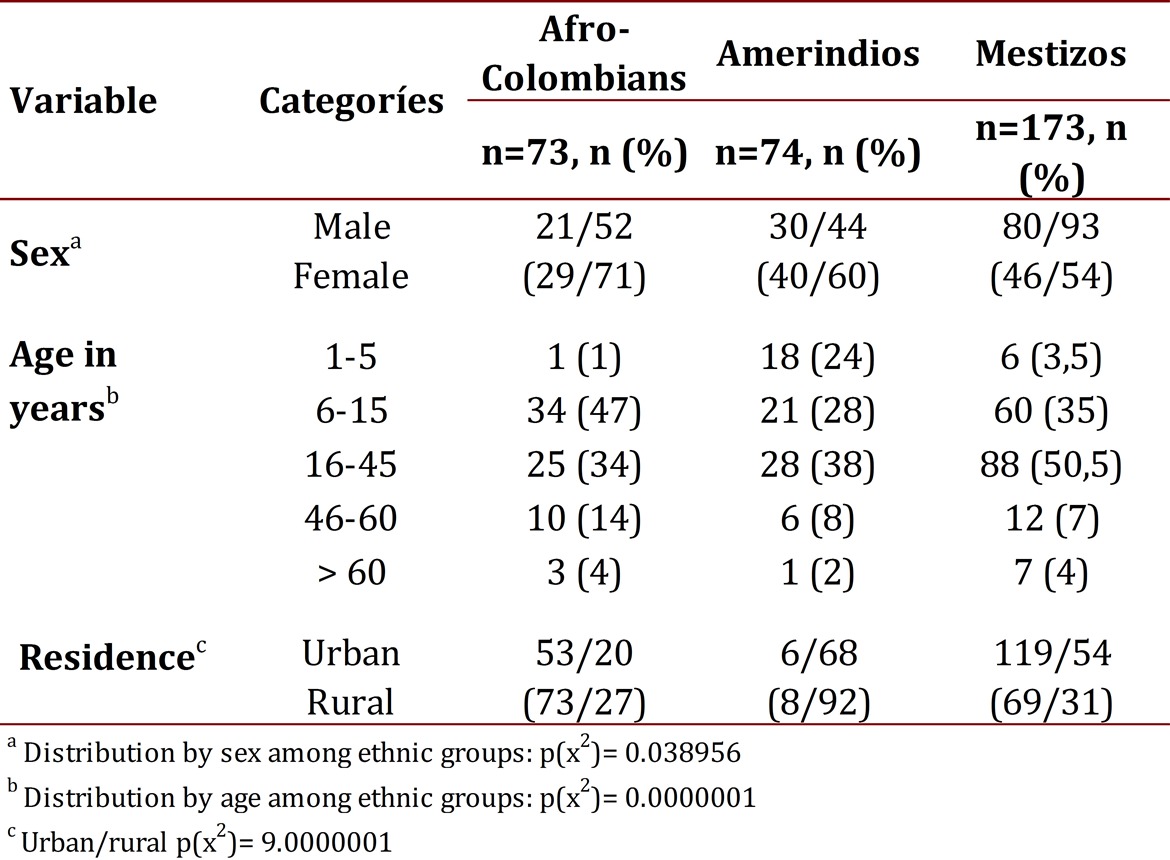
Characteristics of the study population by ethnicity. The probability value (p) for the relationship of each analyzed variable (sex, age, residence area) with the ethnicity variable is indicated

### Frequency of infection by Plasmodium: 

Using thick smears the frequency of plasmodial infection was found to be 17% (52/320). Among those with an infection, the frequency of each species was: 62% *P. falciparum* (32/52), 27% *P. vivax* (14/52), 11% were mixed infections from both species (6/52). The parasitemia from *P. falciparum* in the infected group (symptomatic and asymptomatic) had confidence intervals on average of 8,082 at 17,190. By ethnicity, the average parasitemia from *P. falciparum* was around 6,500 to 8,000 rings/mL in Amerindians and Mestizos, and was 12,641 rings/mL in Afro-Colombians, with no statistically significant differences (*p*= 0.110649). Perhaps this finding partly derives from the small size of the groups and notorious variations that are reflected in the high standard deviations (Table 2). The parasitemia from *P. vivax* or mixed types were not counted.

**Table 2:**
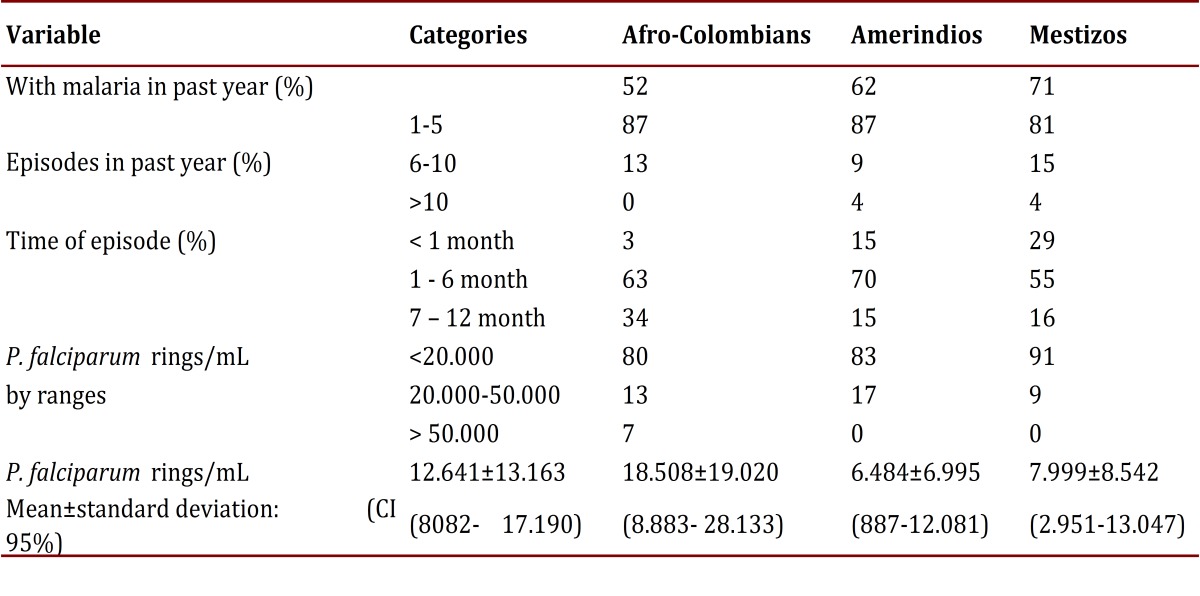
General data on the history of malarial infection during the past year and parasitemia by P. falciparum from the time of study inclusion

Among the 52 cases of *Plasmodium* infection, 33% were observed in asymptomatic individuals (17/52) (Table 3). No significant relationship was found between the species of *Plasmodium* and ethnicity. In total, 65% (207/320) of the population reported a recent history of malarial infection, with an average of two episodes in the last year; 124 individuals reported an episode of malaria in the last 6 months.

**Table 3:**
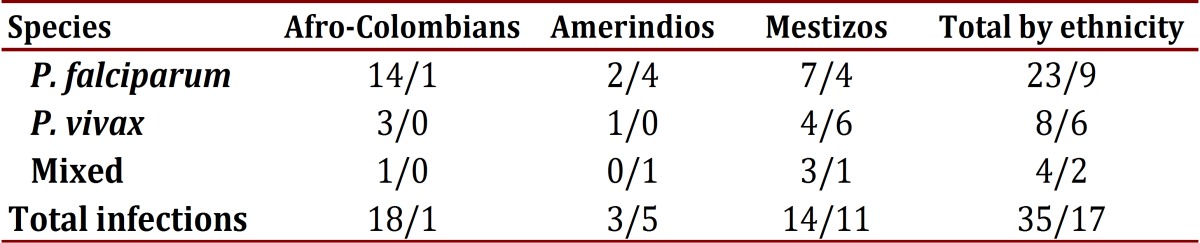
Relationship of symptomatic/asymptomatic infection by ethnicity and species of Plasmodium

### Genotyping of the Duffy blood group: 

The frequency of the T allele at the -46 locus in Afro-Colombians was 0.50; in Mestizos it was 0.91, and was found fixed in Amerindians. The CC and TT genotypes showed frequency of 0.50 in Afro-Colombians, in whom there was an absence of heterozygous; Mestizos presented three genotypes, with infrequent heterozygous. In the 131 locus, the G allele was found less frequently in Afro-Colombians (0.18) and the highest in Amerindians (0.62). Regarding genotype distribution, the lowest frequency of heterozygote's was found in Afro-Colombians (0.25) and the highest was found in Amerindians (0.63). Table 4 details the genotypic and allelic frequencies in each of the populations studied for the loci evaluated.

**Table 4:**
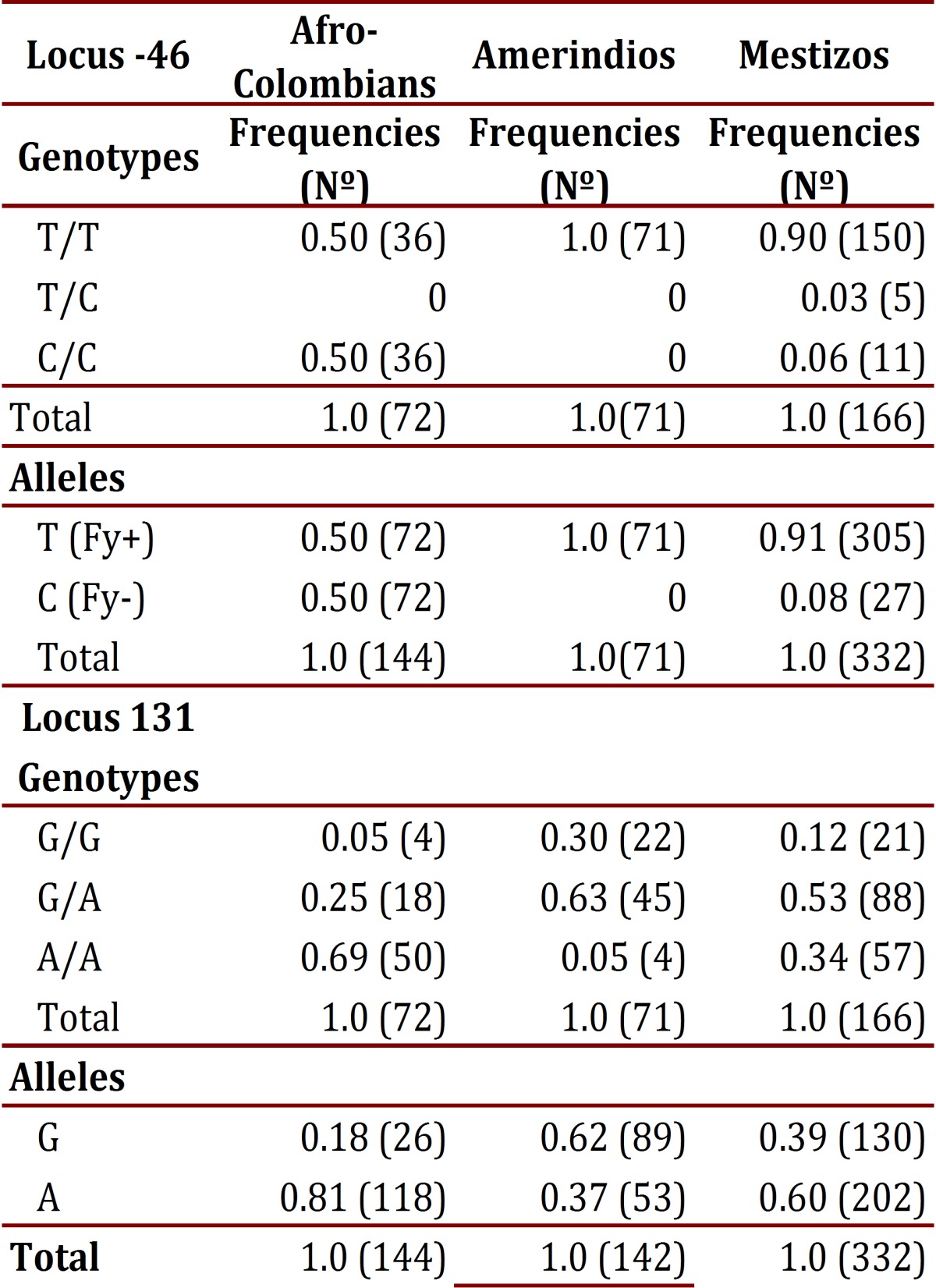
Genotype and allele frequencies of loci -46 and 131 from the Duffy gene by ethnicity

According to neutrality tests of these markers in each of the populations, the -46 locus showed a highly significant difference in frequency among Afro-Colombians and Mestizos (*p* <0.00001) and in the 131 locus it only showed a significant difference among Amerindians (*p*= 0.00402).

The assessment of the genetic structure of populations by means of Wright Fis, Fit and Fst to calculate inbreeding and genetic distance allowed for observation that the Fis values ​​for the loci -46 and 131 in Afro-Colombians show a deficit of heterozygotes in the same way that the -46 locus does for Mestizos. Meanwhile, in the Amerindian and Mestizo populations there is an excess of heterozygotes at locus 131. In evaluating each locus according to the total population, it was observed that in the locus -46 there is a deficit of heterozygotes while in locus 131 there is an excess of them. Regarding the Fst values obtained ​​(> 0.05), it was observed that with locus 131 a medium degree of structuring is achieved; whereas, with locus -46 structuring is very high, >0.25 among the populations. To confirm the above, genetic differences were compared through a tree "Neighbor Joining" (NJ) and the genetic distance of Cavalli-Sforza. A high degree of genetic differentiation was obtained among the three populations. The greatest distance was found between Afro-Colombians (AA) and Amerindians (AM) (0.44). This means that the gene flow between these two ethnic groups (mixed) is the smallest in the population structure of La Italia. The shortest distance was found between Amerindians and Mestizos (0.092), which indicates that the mixed blood subpopulation comes mainly from a mix with Amerindians: the distance of 0.28 between AA and Mestizos (MZ) is almost half that found between AA and AM.

**Figure 1:**
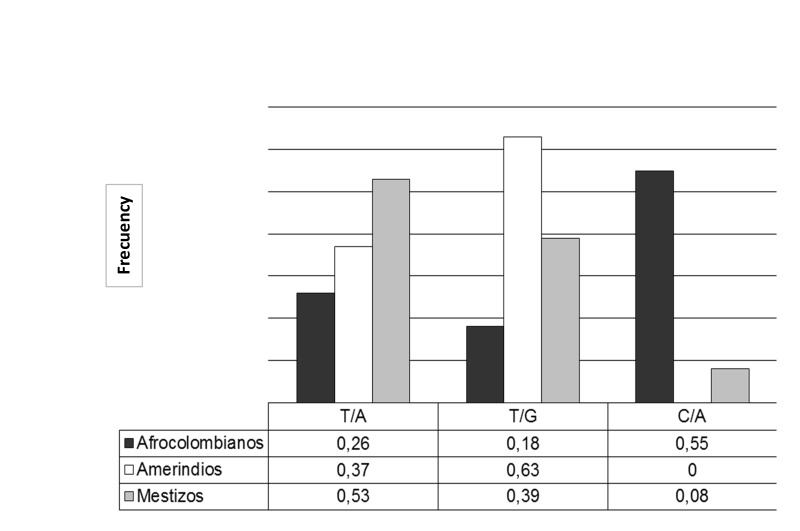
-46/131 Haplotype frequencies of Duffy gene with sta tistical significance (p (chi square) p <0.001) in Afro-Amerindians and mestizos. Comparison of the 3 most probable haplotypes ge nerated Harlequin program.

Additionally, on analyzing the association between ethnicity and genotype at the loci -46 and 131, a highly significant difference (*p* <0.01) was observed due to Afro-Colombians predominantly presenting with the genotype C-46C. This is contrary to what happened in Amerindians and Mestizos who were predominantly G131A genotype. On the other hand, the Amerindians exhibited a frequency 1-5 times greater of G131G while Mestizos were 1-3 times more frequently found with A131A (Fig. 1).

In comparing diplotypes and haplotypes (Table 5), a statistically significant difference was found (*p* <0.001) in the distribution of frequencies in the T/T diplotype: G/A predominated in Amerindians and Mestizos; on the diplotype C/C, A/A was found notably higher in Afro-Colombians, infrequent in Mestizos, and absent in Amerindians; while in C/T, A/A and C/T, G/A were absent or with very low frequency in the three populations.

**Table 5:**
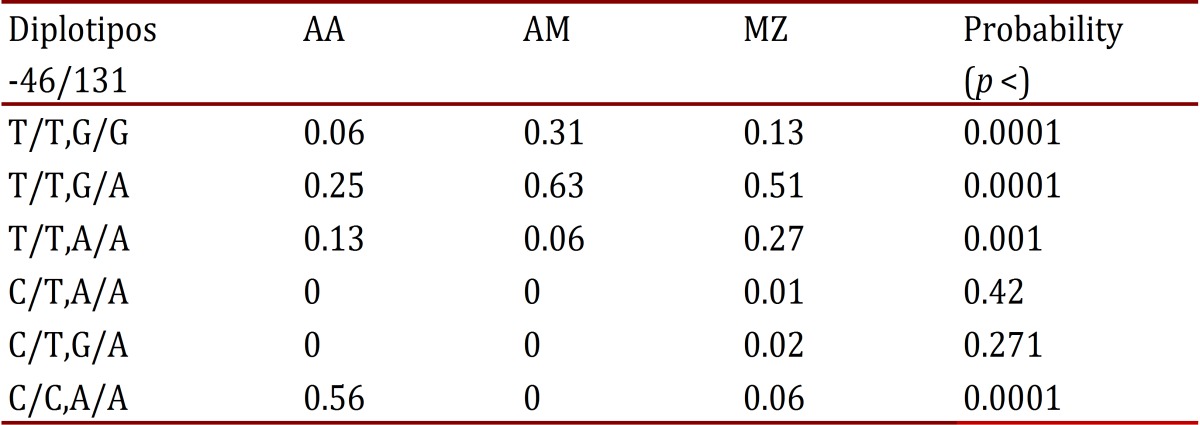
-46/131 Diplotipos frequencies of Duffy gene in Amerin dians (AM), Afro (AA) and mestizos (MZ). Results are highlighted statistically significant

Finally, the comparison of the haplotypes most likely allowed for the finding of three with statistically significant differences among the populations (*p* <0.0001) (Fig. 1). The TG haplotype had the highest frequency in Amerindians and the least in Afro-Colombians; while the CA had the highest frequency in Afro-Colombians, very low in Mestizos and absent in Amerindians. The TA haplotype was predominately found in Mestizos.

**Analysis of association of some variables -** On comparing the average number of episodes of malaria in the last year by diplotype and ethnicity, there was no statistically significant difference. However, on analyzing the number of malaria episodes in the same period according to ethnicity, significant differences were found in Mestizos who had a higher average number of episodes of malaria in the last year (173 vs. 73 Colombians and 74 Amerindians, *p* <0.05).

**Figure 2:**
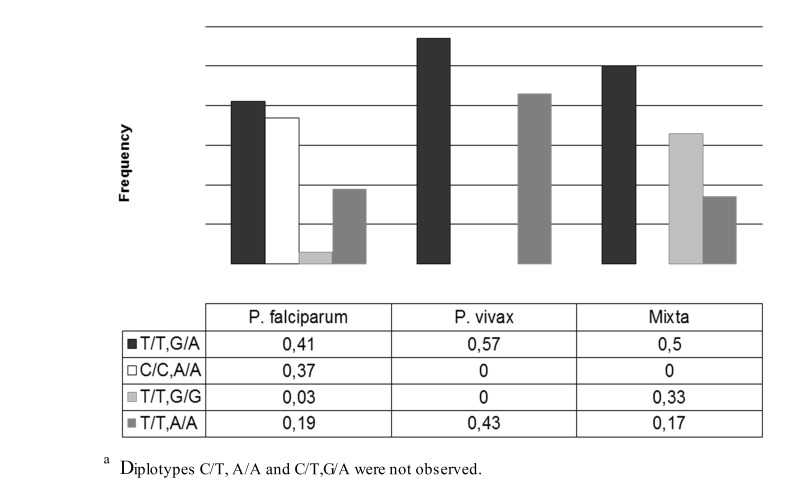
Diplotype frequencies of the Duffy gene and relation to Plasmodium. a Individuals with diplotype C/C, A/A only suffer infection by P. falciparum, p. (chi square) = 0.003

Also, statistical significance was also found in relating diplotype with ethnicity (*p*= 0.003). In individuals with the diplotype C/C, only A/A suffered infection by *P. falciparum. * Individuals with other diplotypes could be infected by *P. vivax* or *P. falciparum* (Fig. 2). No statistically significant differences were found in relation to sex and age with the positive or negative result of the thick smear (*p*= 0.054 and *p*= 0.092).

## Discussion

The present study found that more than one-third of the samples with positive parasitemia were from individuals asymptomatic for malaria. Studies in Colombia have reported values ​​of asymptomatic infection at 0% in schoolchildren from Quibdó^17^ and 14.6% in the population of Tierralta, Cordoba (2-76 years)^24^. The diversity in the population as in the case of age and ethnic composition^25^ could influence the presentation of asymptomatic parasitemia. Nonetheless, at least in the present study, no significant association was found between age and frequency of malarial infection in the differing ethnic groups, so that the relevance of the interaction between age-ethnicity-asymptomatic infections should be more widely explored.

A predominance of the C allele at locus -46 (Fy-) in Afro-Colombians was observed, but less frequently than those reported in African populations^11^ ^,^ ^26^. Other investigators have reported low frequency of this allele in Colombian Mestizo populations^15^. This is probably due to the Afro- Colombian population has suffered a significant level of miscegenation. Further analysis of the genotypes of locus 46 confirmed that Afro-Colombian and mixed ethnicity are not in the equilibrium of Hardy Weinberg. In Afro-Colombian ethnic group there is an excess of homozygous T/T and C/C, which does not support the hypothesis that the C/C genotype is being selected by *P. vivax*, as it has been observed in other populations from Chocó^27^.

The frequency of the G allele in locus 131 predominates in Amerindians, while the frequency of the A allele predominates in Afro-Colombians and Mestizos. Similar data have been reported in individuals from North America (Caucasian-Americans), African-Americans, Turkish individuals and Colombians, where the A131 allele predominates, in contrast to Asian-Americans and Hispanic-American individuals in general in which the G allele predominates^15^ ^,^ ^26^.

The relationship found between ethnicity and the Duffy genotype in position 131 is probably due to Amerindians and Mestizos predominantly carrying the GA genotype, but Afro-Colombians mainly carrying the AA genotype. These data differ from studies of the different ethnic groups in Papua, New Guinea where it was reported that the vast majority of individuals were GG. The Afro-Colombian and Mestizo ethnic groups showed the Hardy Weinberg equilibrium at locus 131; a balance that has also been found in other locations in Turkey^28^. By contrast, the equilibrium of Hardy Weinberg was not detected in Amerindians as a consequence of excess heterozygotes.

The findings of Hardy Weinberg disequilibrium at the locus -46 in the Afro-Colombian and Mestizo populations, and in locus 131 of the Amerindian population could be explained by the presence of inbreeding in each of the three populations and by their migratory history. Fis values ​​for the loci -46 and 131 in Afro-Colombians show a deficit of heterozygote's, which could be explained by a historical review of the Afro-Colombian population migration to this locality: according to oral communication collected at the site. According to this local report, the Afro-Colombian population originally came from two localities - some individuals from the south of Chocó and others from the northwest. This phenomenon could explain the absence of heterozygotes in La Italia, which was only fairly recently established (i.e. 1927). Similarly, the disequilibrium that exists in -46 loci in the Mestizo population, in which a deficit of heterozygote's is also observed, could be explained by the colonization of Antioquian populations and, in recent years, by the displacements from areas farther east, i.e. Caquetá, Putumayo and Amazon areas of Colombia. In contrast, disequilibrium only exists in locus 131 for the Amerindian population due to an excess of heterozygotes.

The high values ​​of Fst are showing that there is structuring by ethnic origin among the three populations. Therefore, the populations differ genetically from one another using these markers. It should be noted that the highest Fst values ​​are found in the -46 locus, i.e., this marker points out the greatest differences in ethnicity, which corroborates its usefulness as a marker of ethnicity in these populations. Additionally, evaluating the population structures by means of the genetic distance of Cavalli-Sforza confirms, in general, a high degree of genetic differentiation among populations of the three ethnic groups from the same geographical area. The greatest difference is between Amerindians and Afro-Colombians and the least genetic distance is between Amerindians and Mestizos. Additionally, the distance between Amerindians and Mestizos is less than that observed among Amerindians and Afro-Colombians, meaning that Mestizos have a bit more of the Amerindian component than do Afro-Colombians.

The above results indicate that although these populations are located in the same geographical area, there is a persistence of ethnic structure that reflects a high degree of inbreeding yet discrimination among the populations.

This is the first Colombian study on malaria and Duffy blood groups in which comparisons are made of the -46/131 diplotypes. Since there is evidence that the genotypes C-46C and A131A protect against infection by *P. vivax*, it is likely that haplotype C/A serves as the protector in the populations studied since it occurs most frequently in the Afro-Colombian population.

By associating Duffy diplotypes with ethnicity and the infecting species of *Plasmodium*, it was observed that individuals with diplotypes C/C, A/A only suffer infection by *P. falciparum*, while individuals with the other diplotypes can be infected by *P. vivax* or *P. falciparum*. This confirms that this is the protector diplotype against *P. vivax*. However, the low frequency of infections by this species prevented the obtaining of significant results regarding the association between ethnicity and species, although *P. vivax* predominates in Mestizos and *P. falciparum* predominates in Afro-Colombians. These results differ from those reported by Montoya et al, who noted a predominance of infection by *P. vivax *in the Emberá tribe of Amerindians and by *P. falciparum* in the Kuna tribe of Amerindians^16^.

The results deom the Duffy genotype and of this research confirm that, as in the region of the Brazilian Amazon^29^ no FY- individual was found with infected by *P. vivax*. This contradicts that which was reported in Colombia in 1994^16^ and the latest reports from several countries in Africa^5^ ^,^ ^6^. One explanation for the difference in results in the Colombian context may lie in the technical limitation of the serological technique for detecting the weak Fyx phenotype, as well as in the existence of different genetic variants of *P. vivax*. These phenomena were not explored in this study. Future work in Colombia should clarify associations between infection from different variants of *P. vivax* and the Duffy genotypes by applying techniques based on nucleic acid amplification by chain reaction of polymerase in addition to assessing the size of fragments after digestion with restriction enzymes (RFLP) or nucleic acid sequencing.
